# Circular RNA Circ_0013958 Functions as a Tumor Promoter in Ovarian Cancer by Regulating miR-637/PLXNB2 Axis

**DOI:** 10.3389/fgene.2021.644451

**Published:** 2021-07-21

**Authors:** Yanfei Liang, Kaiyi Meng, Rui Qiu

**Affiliations:** Department of Gynecology, The Second Nanning People's Hospital, Nanning, China

**Keywords:** circ_0013958, miR-637, PLXNB2, ovarian cancer, tumor growth

## Abstract

**Background:** Circular RNAs (circRNAs) have emerged as important regulators in diverse human malignancies, including ovarian cancer (OC). This study was performed to explore the function and regulatory mechanism underlying circ_0013958 in OC progression.

**Methods:** Quantitative real-time polymerase chain reaction (qRT-PCR) or Western blot assay was applied to examine the expression of circ_0013958, microRNA-637 (miR-637), and Plexin B2 (PLXNB2). The target relationship between miR-637 and circ_0013958 or PLXNB2 was verified by dual-luciferase reporter assay or RNA immunoprecipitation (RIP) assay. Cell Counting Kit-8 (CCK-8) and colony formation assays were employed to detect cell viability and clonogenicity ability, respectively. Cell migration and invasion were analyzed by Transwell assay. Cell apoptosis was monitored by flow cytometry. The role of circ_0013958 *in vivo* was determined by xenograft tumor assay.

**Results:** Circ_0013958 and PLXNB2 were upregulated, while miR-637 was downregulated in OC tissues and cells. Circ_0013958 acted as a sponge for miR-637 to regulate the expression of PLXNB2 in OC cells. The repression effects of circ_0013958 knockdown on cell proliferation, migration, invasion, and apoptosis in OC cells were partly attenuated by the miR-637 inhibitor. And miR-637 targeted PLXNB2 to suppress OC cell proliferation, migration, and invasion. Moreover, circ_0013958 silencing blocked OC tumor growth *in vivo*.

**Conclusion:** Circ_0013958 knockdown impeded OC development through modulating the miR-637/PLXNB2 axis, highlighting a therapeutic target for OC.

## Introduction

As the most frequent gynecological tumor, ovarian cancer (OC) attacked about 300,000 people and resulted in 180,000 cases of deaths in 2018 (Jayson et al., 2014; Bray et al., 2018). Due to the deficiency of effective diagnostic biomarkers, the 5-year survival rate of patients with advanced OC is only ~25% (Timmermans et al., 2018). Though the standard therapy works, OC is still easy to recur (Hennessy et al., 2009). Therefore, developing novel biomarkers and clarifying the underlying molecular mechanisms of OC are extremely urgent.

Circular RNAs (circRNAs) are a class of non-coding RNAs (ncRNAs) with special covalently closed-loop structure and are closely related to the aggressive development of various malignancies, including OC (Lyu and Huang, 2017; Patop and Kadener, 2018). Moreover, circRNAs could function as biomarkers of human cancers for their high stability and specific expression (Su et al., 2019). For example, serum circSETDB1, circ-ABCB10, and serum circMAN1A2 could be used as a promising biomarker for the progression of OC (Chen et al., 2019; Fan et al., 2019; Wang W. et al., 2019). Accumulating evidence indicates that circRNAs have great impact on OC development (Feng et al., 2019). Derived from acid phosphatase 6 (ACP6), circ_0013958 (Location: chr1:147131074-147131890; Spliced sequence length: 340 bp) was reported to facilitate the development of OC (Pei et al., 2020). However, the molecular mechanism by which circ_0013958 affected OC progression had not been elucidated.

MicroRNAs (miRNAs) are also non-coding molecules, ~22 nucleotides long, which could directly bind to the 3′ untranslated region (3′UTR) of target mRNAs so as to inhibit their expression and functions (Ambros, 2004; Huntzinger and Izaurralde, 2011). Abundant miRNAs were corroborated to have clinical relevance and regulatory effects on OC progression (Kafshdooz et al., 2018). MiR-320 is highly associated with the metastasis of OC, which could indicate unfavorable prognosis of OC (Wang et al., 2017). MiR-552 contributed to the development of OC by targeting PTEN (Zhao et al., 2019). Inversely, miR-128 acted the tumor-suppressor role in OC by targeting HOXB8 (Li et al., 2019). MiR-637 expression was downregulated in OC and was involved in the circ_0051240-mediated promotion of OC development (Zhang M. et al., 2019). As a potential target miRNA of circ_0013958 predicted by Circular RNA Interactome, the association between miR-637 and circ_0013958 in OC remains to be investigated.

Plexin B2 (PLXNB2) was proved to function in post-natal neurogenesis and affect the migration of subventricular zone (SVZ)-derived neuroblasts (Saha et al., 2012). PLXNB2 exerted osteogenesis promoting role in human bone marrow mesenchymal stem cells (BMSCs) through activating RhoA signaling (Zhang Y. et al., 2019). Furthermore, PLXNB2 was identified to be a prognostic biomarker for glioma and facilitated to glioma invasion and vascularization (Le et al., 2015). In OC, PLXNB2 expression was upregulated. And silencing of PLXNB2 inhibited OC cell proliferation and invasion (Xiang and Cheng, 2018). Herein, PLXNB2 was predicted to be a target mRNA of miR-637 by Targetscan. We further studied the co-effects of PLXNB2 and miR-637 on OC development.

In this project, the significant upregulation of circ_0013958 in OC tissues and cells was detected. The functional impact of circ_0013958 on OC cell proliferation, migration, invasion, and tumorigenesis was explored, as well as the involvement of regulatory axis circ_0013958/miR-637/PLXNB2 in OC progression.

## Materials and Methods

### Tissue Collection and Cell Culture

Thirty pairs of OC tissues and neighboring non-cancerous tissues were procured from OC patients who had received ovariectomy at The Second Nanning People's Hospital. Before surgery, we obtained approval from the Ethics Committee of The Second Nanning People's Hospital and written informed consent from these 30 OC patients.

Normal human ovarian surface epithelial cell line (HOSE; #7310, ScienCell Research Laboratories, Carlsbad, CA, USA) and OC cell lines SKOV3 (ATCC® HTB-77, American Type Culture Collection, Manassas, VA, USA) and CAOV3 (ATCC® HTB-75) were maintained in a Roswell Park Memorial Institute (RPMI)-1640 medium (Gibco, Grand Island, NY, USA), added with 10% fetal bovine serum (FBS; HyClone, Logan, UT, USA) and 1% penicillin/streptomycin (Sigma-Aldrich, St. Louis, MO, USA) at 37°C in the presence of 5% CO_2_.

### Cell Transfection

Small interfering RNA (siRNA) targeting circ_0013958 (si-circ_0013958, 5′-GTGTCAGAAAGAAGGTAGAGT-3′) and its negative control si-NC (5′-AACAGTCGCGTTTGCGACTGG-3′) was procured from GENEWIZ (Suzhou, China). MiR-637 mimic (miR-637) and mimic control (miR-NC), as well as miR-637 inhibitor (in-miR-637) and negative control (in-miR-con), were all supplied by RIBOBIO Co. Ltd. (Guangzhou, China). The overexpression vector of PLXNB2 (pcDNA-PLXNB2) was constructed by inserting its cDNA sequence into the pcDNA 3.1 vector (Invitrogen, Carlsbad, CA, USA) (pcDNA-con). Aforementioned oligonucleotides or vectors were introduced into SKOV3 and CAOV3 cells using Lipofectamine 3000 (Invitrogen) referring to the producer's guidance.

### Quantitative Real-Time Polymerase Chain Reaction (qRT-PCR)

After isolation from clinical tissues or cells using the TRIzol Reagent (Invitrogen), total RNA was subjected for complementary DNA (cDNA) synthesis with the aid of M-MLV reverse transcriptase (Beyotime, Shanghai, China) or miRNA First-Strand cDNA Synthesis Kit (Agilent, Santa Clara, CA, USA). Following qRT-PCR was implemented utilizing the SYBR Master Mix (Applied Biosystems Inc., Foster City, CA, USA) or the miRNA-specific TaqMan MiRNA Assay Kit (Applied Biosystems Inc.) on the ABI PRISM 7500 real-time PCR System (Applied Biosystems Inc.). Relative expression was calculated using the 2^−ΔΔCt^ formula, with glyceraldehyde-3-phosphate dehydrogenase (GAPDH, for circ_0013958 and PLXNB2) or U6 (miR-637) as an internal reference. All primers involved in the qRT-PCR assay are listed in Table 1.

**Table 1:** The primer sequences for qRT-PCR assay in this study.

**Gene**	**Sequence**	
	**Forward (5′-3′)**	**Reverse (5′-3′)**
circ_0013958	5′-TTCAACCCACAGGAGGTCTT-3′	5′-ATAGCTGGGGGTTCCACTCT-3′
miR-637	5′-ACTGGGGGCTTTCGGGCT-3′	5′-GAACATGTCTGCGTATCTC-3′
PLXNB2	5′-CTTCAGCCTGATCCAGAGGTTTG-3′	5′-GTGGAACACGTAGTCTGTACCC-3′
GAPDH	5′-TGTTCGTCATGGGTGTGAAC-3′	5′- ATGGCATGGACTGTGGTCAT-3′
U6	5′-CTCGCTTCGGCAGCACA-3′	5′-AACGCTTCACGAATTTGCGT-3′

### Nuclear-Cytoplasmic Fractionation Assay

This assay was executed to clarify the subcellular location of circ_0013958. As the user's manual directed, total RNA was extracted from cytoplasmic and nuclear fractions exploiting the Cytoplasmic & Nuclear RNA Purification Kit (Norgen Biotek, Thorold, ON, Canada). GAPDH and U6 acted as the positive control for cytoplasmic and nuclear fractions, respectively. The proportion of circ_0013958 in cytoplasm and nucleus of OC cells was evaluated by the qRT-PCR assay.

### Dual-Luciferase Reporter Assay

Circular RNA Interactome (https://circinteractome.nia.nih.gov/index.html) and Targetscan (http://www.targetscan.org/mamm_31/) were searched to predict the potential targets of circ_0013958 and miR-637, respectively. Wild-type luciferase reporters of circ_0013958 (WT_circ_0013958) and PLXNB2 3′-UTR (WT-PLXNB2 3′-UTR) were established by cloning the fragment sequence harboring binding sites with miR-637 into the pGL3 luciferase promoter vector (Promega, Madison, WI, USA). Likewise, mutant-type ones (MUT_circ_0013958 and MUT-PLXNB2 3′-UTR) were constructed by inserting partial sequence embracing the mutant binding sites. Afterward, the constructs and miR-NC or miR-637 were cotransfected into SKOV3 and CAOV3 cells. At 48 h after transfection, a Dual-Luciferase Reporter Assay System (Promega) was used for measurement of luciferase activity, following the manufacturer's direction.

### RNA Immunoprecipitation (RIP) Assay

The RIP assay was exploited to validate the target relationship between circ_0013958 and miR-637 in accordance with the user's manual of the EZ-Magna RIP Kit (Millipore, Billerica, MA, USA). In brief, SKOV3 and CAOV3 cells were lysed in a specific lysis buffer and then incubated with a RIP buffer containing magnetic beads, which were conjugated with anti-IgG (ab109489; Abcam, Shanghai, China) or anti-AGO2 antibody (ab32381; Abcam). Subsequently, precipitated RNA was isolated and subjected for the qRT-PCR assay to evaluate the abundance of circ_0013958 and miR-637.

### Cell Counting Kit-8 (CCK-8) Assay

CCK-8 was conducted to assess the cell viability of SKOV3 and CAOV3 cells. After transfection for the indicated time, 5 × 10^3^ OC cells in a 100 μl complete medium were plated in 96-well plates, and then a 10 μl CCK-8 reagent (Sigma-Aldrich) was pipetted into each well. After an additional 2 h, a microplate reader (Bio-Rad, Hercules, CA, USA) was utilized to record the optical density (OD) of each well at 450 nm.

### Colony Formation Assay

Transfected SKOV3 and CAOV3 cells (6 × 10^2^) were seeded in six-well plates and then maintained at 37°C. Twelve days later, generated cell colonies (containing exceeding 50 cells) were subjected for fixation with 4% paraformaldehyde (Sigma-Aldrich) and staining using crystal violet (Solarbio, Beijing, China), followed by counting under a dissecting microscope.

### Transwell Migration and Invasion Assays

Transwell chambers (8-μm size, BD Biosciences, San Jose, CA, USA) precoated with Matrigel (BD Biosciences) or not were utilized to detect cell invasion or migration of OC cells, respectively. Transfected SKOV3 and CAOV3 cells in a medium without FBS were placed into the upper chambers. Meanwhile, a complete medium was added into the lower ones. Twenty-four hours later, OC cells invaded or migrated through the fibronectin-coated polycarbonate membrane were immobilized by 4% paraformaldehyde and dyed using crystal violet, and then counted using a microscope (×100).

### Flow Cytometry

Here, the Annexin V-fluorescein isothiocyanate (FITC) Apoptosis Detection kit (BD Biosciences) was applied to determine the cell apoptosis of SKOV3 and CAOV3 cells. In brief, transfected OC cells (1 × 10^5^) were collected and resuspended in a specific binding buffer. Then Annexin V-FITC and propidium iodide (PI) solution were added in succession to stain cells in the dark. In the end, the apoptotic cells were monitored exploiting a flow cytometer (BD Biosciences) and computed using the following formula: apoptosis rate = apoptotic cells/total cells × 100%.

### Western Blot Assay

The current assay was carried out based on the procedures reported previously (Lu et al., 2020). Briefly, 40 μg protein was loaded on fresh sodium dodecyl sulfate polyacrylamide gel electrophoresis (SDS-PAGE) and then electro-transferred onto the polyvinylidene fluoride membranes (Millipore). The membranes were subjected for blockage in 5% fat-free milk, incubation with primary antibody against PLXNB2 (ab229950; Abcam) or GAPDH (ab9485; Abcam) and secondary antibody (ab205718; Abcam), followed by band visualization using the chemiluminescence (ECL) detection system (Millipore). At last, the gray value was analyzed by Image J software (NIH, Bethesda, MD, USA).

### Xenograft Tumor Assay

Animal experiments in this study were permitted by the Ethics Committee of The Second Nanning People's Hospital. Five-week-old BALB/c nude mice (female) obtained from Sebiona (Guangzhou, China) were subcutaneously inoculated with 2 × 10^6^ SKOV3 cells stably expressing sh-NC or sh-circ_0013958 (*n* = 6). The size of formed tumors was recorded every 7 days (0.5 × length × width^2^). Twenty-eight days later, all mice were sacrificed. Xenograft tumors were collected for weighing and detecting the expression of circ_0013958, miR-637, and PLXNB2.

### IHC Staining Assay

Xenograft tumors were fixed with 10% formaldehyde and embedded in paraffin, after incubating the sections with 5% goat serum for 30 min at room temperature. Besides, the sections were incubated with PLXNB2 antibody (ab229950; Abcam) overnight at 4°C and with HRP labeled secondary antibody (ab205718; Abcam) for 30 min at room temperature. The sections were then visualized using diaminobenzidine (DAB) and hematoxylin, and the PLXNB2 positive cells were observed under a microscope.

### Statistical Analysis

All data in this study were from more than three independent experiments and processed utilizing SPSS 21 (IBM Corp., Armonk, NY, USA). Data were shown as mean ± standard deviation (SD). Statistical difference was compared with Student's *t*-test or analysis of variance (ANOVA) followed by Tukey's test. The correlation between circ_0013958 and miR-637 in OC tissues was assessed by Pearson's correlation analysis. Receiver operating characteristic (ROC) analysis was employed to evaluate the prognostic sensitivity and specificity of circ_0013958 in OC patients. *P* < 0.05 was deemed as statistically significant.

## Results

### Circ_0013958 Was Upregulated, While miR-637 Was Downregulated in OC Tissues and Cells

**Figure 1:**
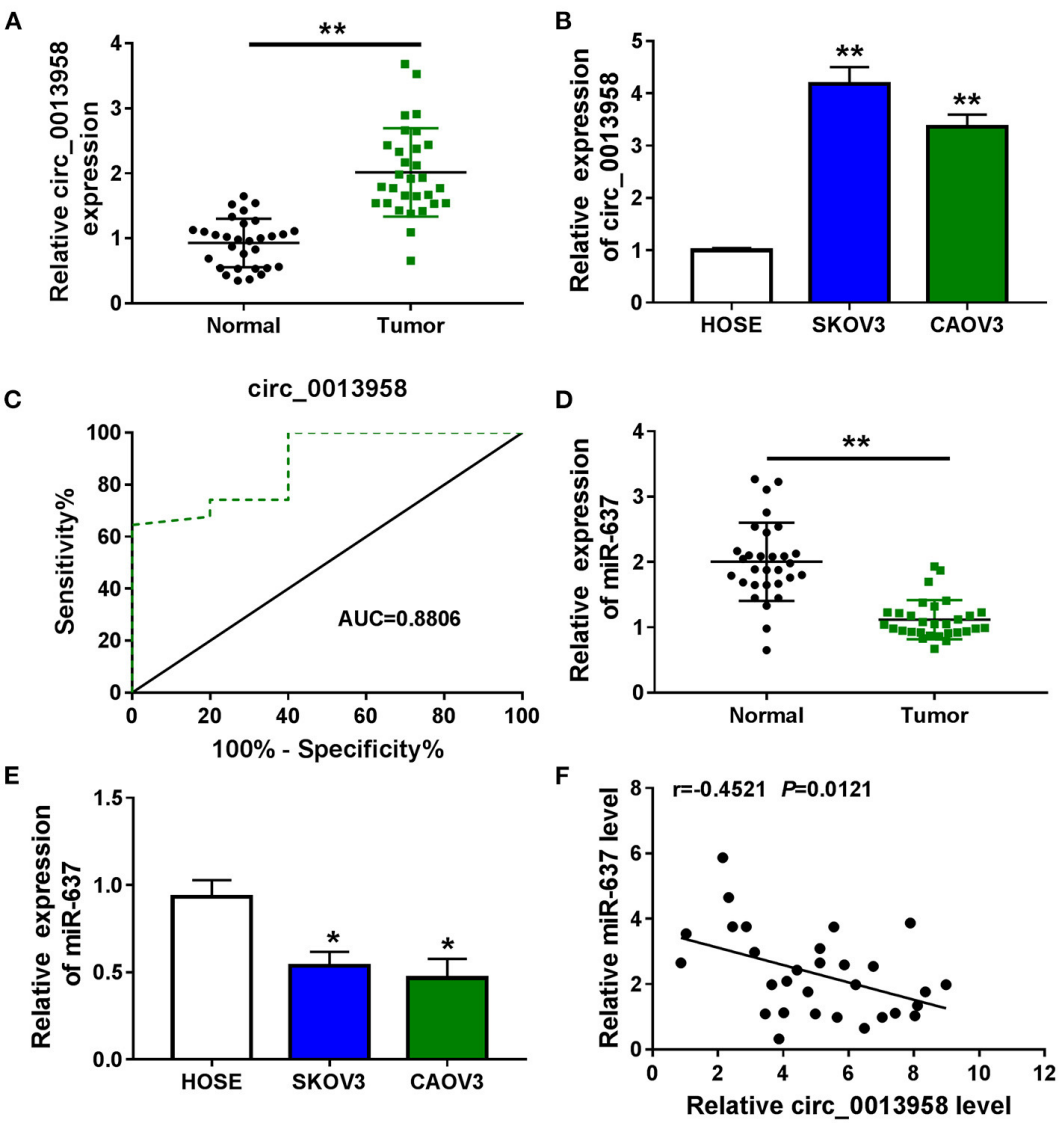
Circ_0013958 was upregulated, while miR-637 was downregulated in OC tissues and cells. **(A)** QRT-PCR assay for the expression of circ_0013958 in OC tissues and adjacent normal tissues (*n* = 30). **(B)** QRT-PCR analysis for the expression of circ_0013958 in OC cell line (SKOV3 and CAOV3) and human ovarian surface epithelial cells (HOSE). **(C)** ROC curve analysis of the prognostic sensitivity and specificity of circ_0013958 in OC patients in accordance with the 5-year survival of OC patients. **(D)** QRT-PCR assay for the expression of miR-637 in OC tissues and matched normal tissues (*n* = 30). **(E)** QRT-PCR assay for the expression of miR-637 in HOSE, SKOV3, and CAOV3 cells. **(F)** Pearson's correlation analysis for the correlation between the expression levels of circ_0013958 and miR-637 in OC tissues (*n* = 30). *P < 0.05; **P < 0.01.

Dysregulated circRNAs were discovered to have an association with cancer treatment (Huang et al., 2019). The expression of circ_0013958 in OC tissues and matched normal tissues was examined by the qRT-PCR assay. Results showed that circ_0013958 was highly expressed in OC tissues (*n* = 30) in contrast to paired normal tissues (*n* = 30) (Figure 1A). And circ_0013958 was highly expressed in OC cells (SKOV3, CAOV3, A2780, and OVCAR-3) relative to HOSE cells (Figure 1B and Supplementary Figure 1). We further analyzed the prognostic accuracy of using circ_0013958 in the diagnosis of OC using receiver operating characteristic (ROC) curves based on the 5-year survival of OC patients. As shown in Figure 1C, the area under the ROC curve (AUC) is 0.8806 (*P* = 0.0070), indicating that circ_0013958 could be used as a prognostic biomarker of OC patients. Moreover, the qRT-PCR assay uncovered the downregulation of miR-637 in OC tissues (Figure 1D) and cells (Figure 1E) when compared to corresponding controls. Furthermore, the expression level of circ_0013958 was negatively correlated (*r* = −0.4521, *P* = 0.0121) with that of miR-637 in 30 cases of OC tissues (Figure 1F). Collectively, circ_0013958 was upregulated, while miR-637 was downregulated in OC tissues and cells.

### Circ_0013958 Could Sponge miR-637 in OC Cells

**Figure 2:**
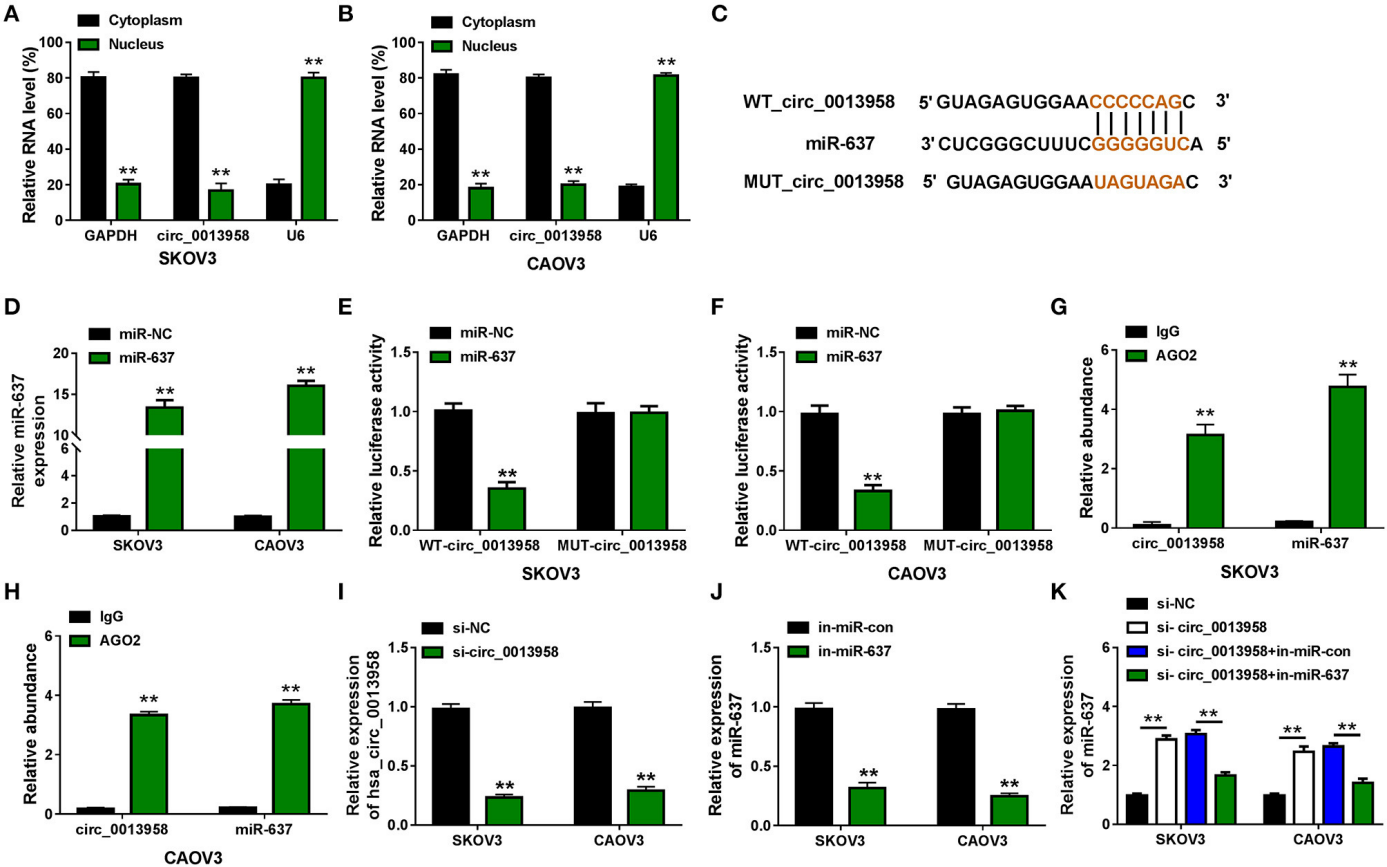
Circ_0013958 acts as a sponge for miR-637 in OC cells. **(A,B)** Nuclear-cytoplasmic fractionation assay for the subcellular location of circ_0013958 in SKOV3 and CAOV3 cells. **(C)** Binding sites between circ_0013958 and miR-637 were predicted by the CircInteractome database. **(D)** QRT-PCR assay for the expression of miR-637 in SKOV3 and CAOV3 cells transfected with miR-NC or miR-637. **(E,F)** Dual-luciferase reporter assay for the luciferase activity of SKOV3 and CAOV3 cells cotransfected with WT-circ_0013958 or MUT-circ_0013958 and miR-NC or miR-637. **(G,H)** RIP assay for the enrichment of circ_0013958 and miR-637 in SKOV3 and CAOV3 incubated with IgG or AGO2 antibody. **(I)** QRT-PCR analysis for the expression of circ_0013958 in SKOV3 and CAOV3 cells transfected with si-NC or si-circ_0013958. **(J)** QRT-PCR analysis for the expression of miR-637 in SKOV3 and CAOV3 cells transfected with in-miR-con or in-miR-637. **(K)** QRT-PCR analysis for the expression of miR-637 in SKOV3 and CAOV3 cells transfected with si-NC, si-circ_0013958, si-circ_0013958 + in-miR-con, or si-circ_0013958 + in-miR-637. **P < 0.01.

Increasing evidence suggested that circRNAs were capable to sponge miRNAs to exert their own functions (Hansen et al., 2013). Here, the nuclear-cytoplasmic fractionation assay was applied to determine the subcellular location of circ_0013958 in SKOV3 and CAOV3 cells. As exhibited in Figures 2A,B, circ_0013958 was mainly predominantly localized in the cytoplasm of the two OC cell lines, implying that circ_0013958 might sponge miR-637 in the cytoplasm. By searching CircInteractome, we found that circ_0013958 (5′-CCCCCAG-3′) harbored the complementary binding sites of miR-637 (5′-GGGGGUC-3′) (Figure 2C). SKOV3 and CAOV3 cells with miR-637 overexpression were established by transfection with miR-637, with miR-NC as the negative control (Figure 2D). Data from the dual-luciferase reporter assay revealed that introduction of miR-637 resulted in more than 60% reduction of the luciferase activity of SKOV3 and CAOV3 cells cotransfected with WT-circ_0013958, while it had no obvious impact on that of cells cotransfected with MUT-circ_0013958 (Figures 2E,F). The RIP assay manifested that both circ_0013958 and miR-637 abundance were highly enriched in the AGO2 group compared to the IgG group (Figures 2G,H). The QRT-PCR assay showed that transfection with si-circ_0013958 apparently reduced the expression of circ_0013958 in SKOV3 and CAOV3 cells in contrast to si-NC (Figure 2I). Introduction of in-miR-637 triggered the significant downregulation of miR-637 in OC cells relative to in-miR-con (Figure 2J). Additionally, circ_0013958 knockdown efficiently upregulated miR-637 expression in SKOV3 and CAOV3 cells, which was weakened by miR-637 inhibition (Figure 2K). Therefore, circ_0013958 could sponge miR-637 and negatively regulate its expression in OC cells.

### Circ_0013958 Knockdown Mediated Proliferation, Migration, Invasion, and Apoptosis of OC Cells by Sponging miR-637

**Figure 3:**
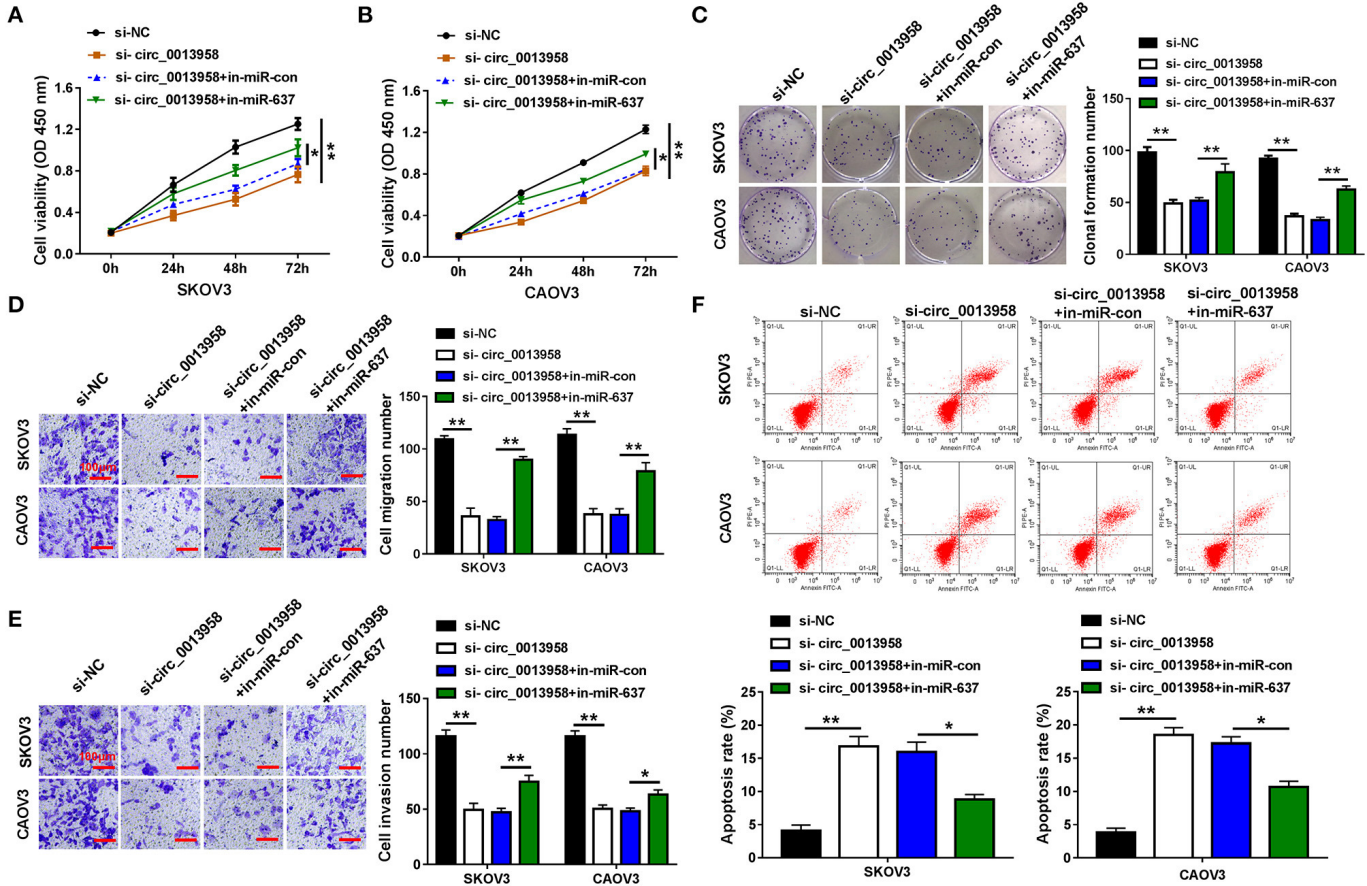
Circ_0013958 knockdown inhibited proliferation, migration, and invasion but promoted apoptosis in OC cells by negatively regulating miR-637. SKOV3 and CAOV3 cells were transfected with si-NC, si-circ_0013958, si-circ_0013958 + in-miR-con, or si-circ_0013958 + in-miR-637, respectively. **(A,B)** CCK-8 assay for the cell viability of transfected cells. **(C)** Colony formation assay for the clonogenicity ability of transfected cells. **(D,E)** Transwell assay for the cell migration and invasion in transfected cells. The scale bar represents 100 μm. **(F)** Flow cytometry for the apoptosis rate of transfected cells. *P < 0.05; **P < 0.01.

As circ_0013958 acted as a sponge for miR-637 in OC cells, we then investigated whether circ_0013958 functions in OC progression by regulating miR-637. When compared with si-NC, circ_0013958 knockdown significantly repressed cell viability of SKOV3 and CAOV3 cells, which was demonstrated by the CCK-8 assay (Figures 3A,B). The colony formation assay testified that depletion of circ_0013958 also inhibited the clonogenicity in OC cells with respect to si-NC (Figure 3C). As shown in Figures 3D,E, the Transwell assay witnessed the circ_0013958 knockdown-induced repressed migration and invasion in SKOV3 and CAOV3 cells with si-NC as the control. Moreover, circ_0013958 deficiency remarkably raised the apoptotic rate of OC cells in contrast to si-NC (Figure 3F). The above-mentioned circ_0013958 knockdown-induced decreased cell viability (Figures 3A,B), clonogenicity (Figure 3C), repressed migration and invasion (Figures 3D,E), and elevated apoptotic rate (Figure 3F) in SKOV3 and CAOV3 cells were all attenuated by miR-637 inhibition. Collectively, miR-637 inhibition could largely reverse the inhibitory effects of circ_0013958 deficiency on proliferation, migration, and invasion of SKOV3 and CAOV3 cells.

### PLXNB2 Was a Target of miR-637 in OC Cells

**Figure 4:**
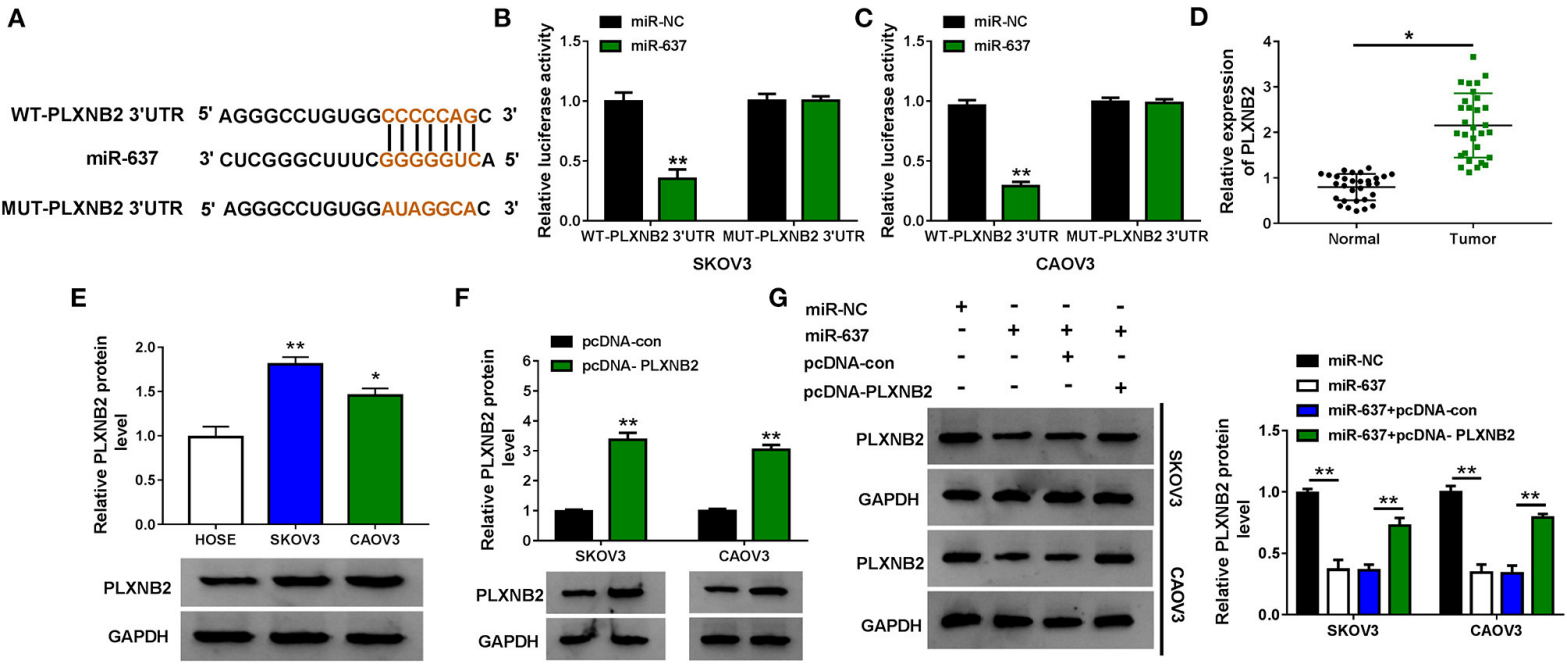
PLXNB2 was a target of miR-637 in OC cells. **(A)** Binding sites between miR-637 and PLXNB2 3′-UTR were predicted by Targetscan. **(B,C)** Dual-luciferase reporter assay for the luciferase activity of SKOV3 and CAOV3 cells cotransfected with WT-PLXNB2 3′-UTR or MUT-PLXNB2 3′-UTR and miR-NC or miR-637. **(D)** QRT-PCR assay for the expression of PLXNB2 mRNA in OC tissues and adjacent normal tissues (*n* = 30). **(E)** Western blot assay for the protein level of PLXNB2 in HOSE, SKOV3, and CAOV3 cells. **(F)** Western blot assay for the protein level of PLXNB2 in SKOV3 and CAOV3 cells transfected with pcDNA-con or pcDNA-PLXNB2. **(G)** Western blot assay for the protein level of PLXNB2 in SKOV3 and CAOV3 cells transfected with miR-NC, miR-637, miR-637 + pcDNA-con, or miR-637 + pcDNA-PLXNB2. *P < 0.05; **P < 0.01.

MiRNA-target mRNA interactions and their regulatory networks have been elucidated to have diverse functions (Kim et al., 2017). Targetscan predicted that PLXNB2 3′-UTR (5′-CCCCCAG-3′) could bind with miR-637 (5′-GGGGGUC-3′) (Figure 4A). Following the dual-luciferase reporter assay verified that the gain of miR-637 markedly suppressed the luciferase density (about 60%) of SKOV3 and CAOV3 cells cotransfected with WT-PLXNB2 3′-UTR, while that of cells cotransfected with MUT-PLXNB2 3′-UTR was changeless (Figures 4B,C). PLXNB2 mRNA and protein levels were upregulated in OC tissues compared to adjacent normal tissues (Figure 4D and Supplementary Figure 2). In addition, the protein level of PLXNB2 in SKOV3 and CAOV3 cells was higher than that in HOSE cells (Figure 4E). SKOV3 and CAOV3 cells with PLXNB2 overexpression were established by transfection with pcDNA-PLXNB2; pcDNA-con acted as the control (Figure 4F). As we can see from Figure 4G, enforced expression of miR-637 evidently repressed the protein level of PLXNB2 in OC cells, while additional pcDNA-PLXNB2 efficiently recovered it. Taken together, miR-637 targeted PLXNB2 and inversely regulated PLXNB2 expression.

### miR-637 Exerted Tumor-Repressor Role by Targeting PLXNB2

**Figure 5:**
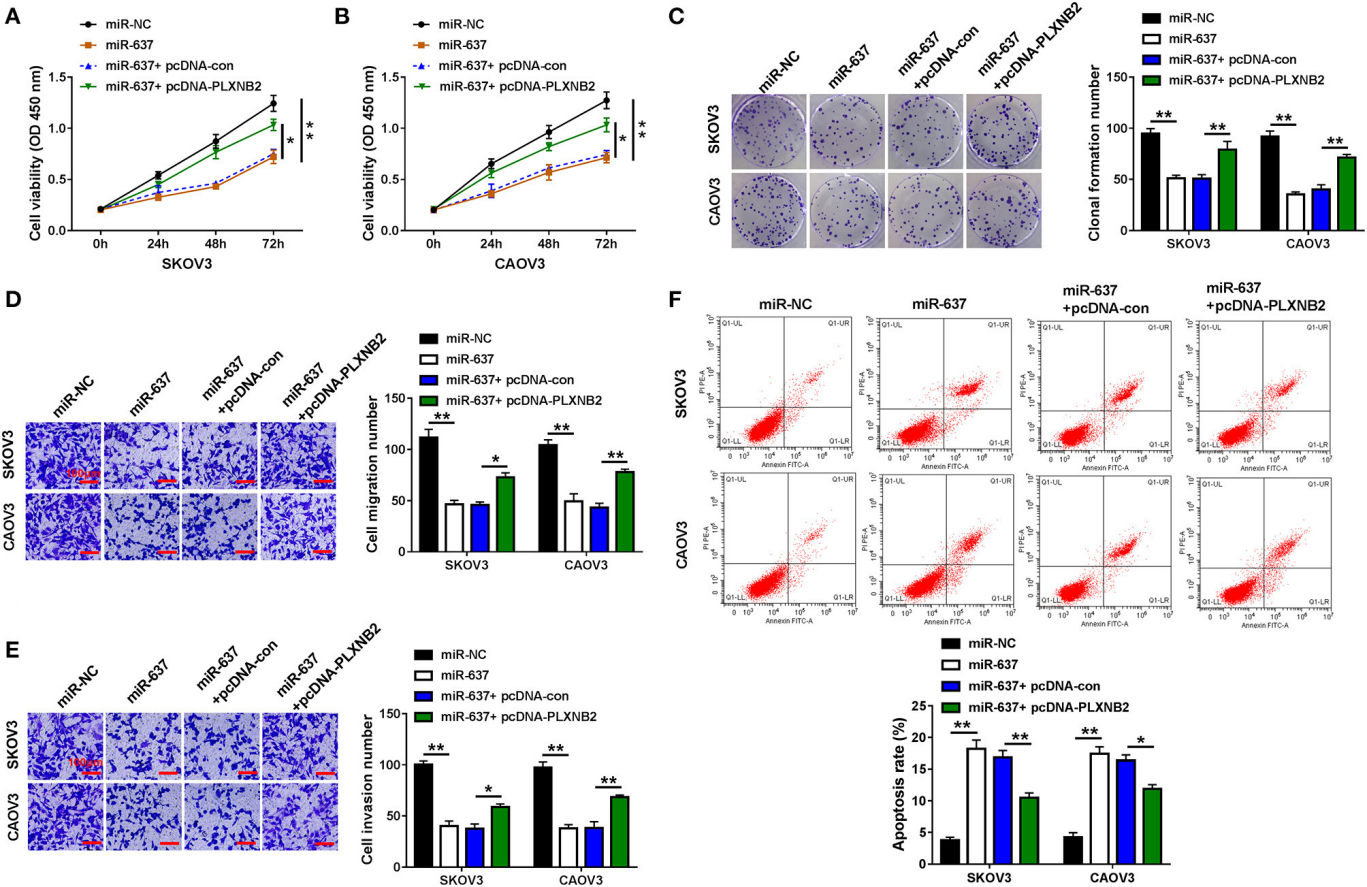
MiR-637 exerted a tumor-repressor role in OC by targeting PLXNB2. SKOV3 and CAOV3 cells were transfected with miR-NC, miR-637, miR-637 + pcDNA-con, or miR-637 + pcDNA-PLXNB2, respectively. **(A,B)** CCK-8 assay for the cell viability of transfected cells. **(C)** Colony formation assay for the clonogenicity ability of transfected cells. **(D,E)** Transwell assay for the cell migration and invasion in transfected cells. The scale bar represents 100 μm. **(F)** Flow cytometry for the apoptosis rate of transfected cells. *P < 0.05; **P < 0.01.

We further explored the functions of miR-637 and PLXNB2 on the cellular behaviors of OC cells. Results suggested that miR-637 conspicuously repressed cell viability (Figures 5A,B), clonogenicity (Figure 5C), as well as migration and invasion (Figures 5D,E), while it facilitated to cell apoptosis (Figure 5F) in SKOV3 and CAOV3 cells, which were all mitigated by PLXNB2 overexpression. To sum up, miR-637 suppressed proliferation, migration, and invasion while contributing to the apoptosis of OC cells by downregulating the PLXNB2 expression.

### Circ_0013958 Positively Regulated PLXNB2 Expression by Absorbing miR-637

**Figure 6:**
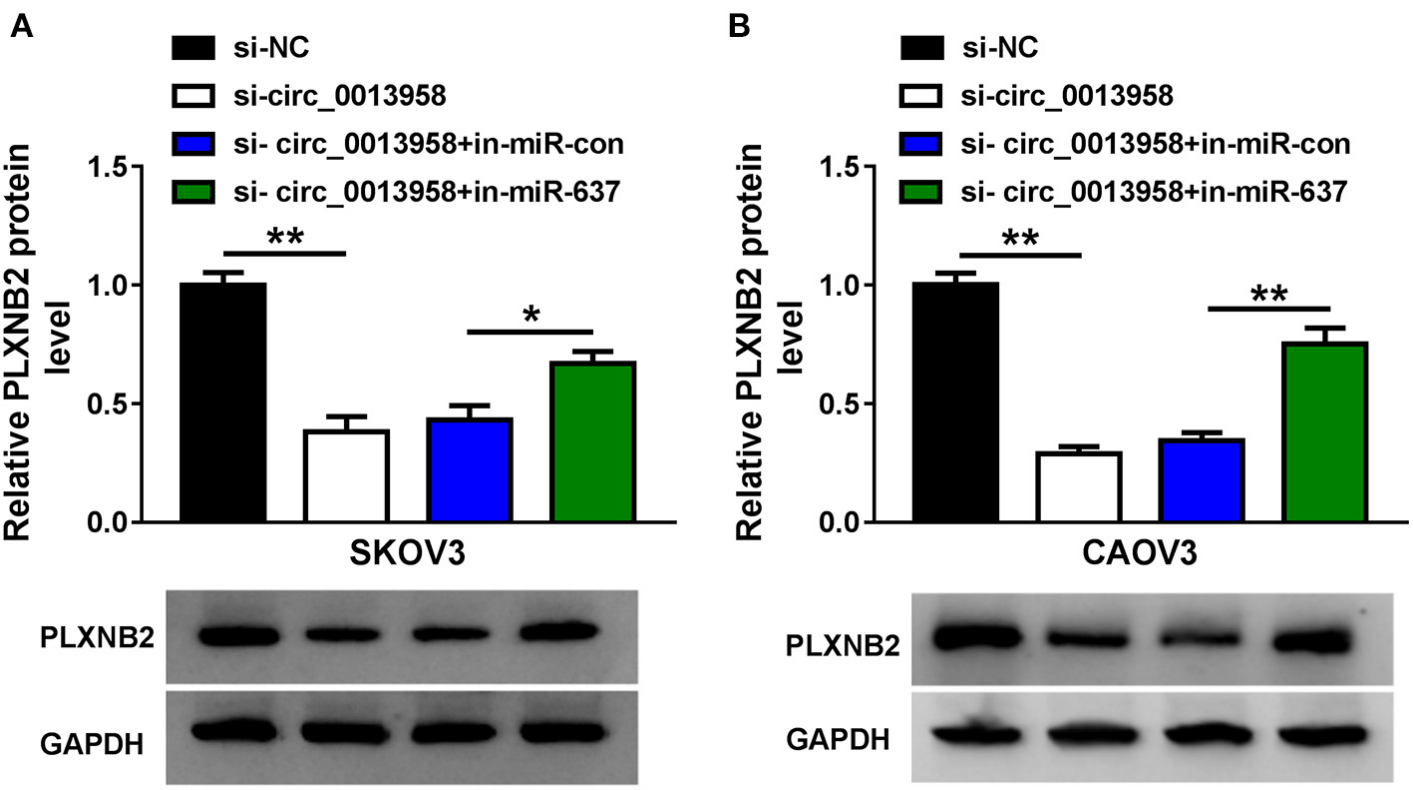
Circ_0013958 positively regulated PLXNB2 expression by absorbing miR-637. **(A,B)** Western blot assay for the protein level of PLXNB2 in SKOV3 and CAOV3 cells transfected with si-NC, si-circ_0013958, si-circ_0013958 + in-miR-con, or si-circ_0013958 + in-miR-637. *P < 0.05; **P < 0.01.

The impact of circ_0013958 on the protein level of PLXNB2 in OC cells was demonstrated by the Western blot assay. Data substantiated that depletion of circ_0013958 downregulated the PLXNB2 protein level, while the introduction of the miR-637 inhibitor reversed the downregulation (Figures 6A,B). And a positive correlation (*r* = 0.5757, *P* = 0.0009) was detected in the levels of PLXNB2 and miR-637 in 30 cases of OC tissues (Supplementary Figure 3). Thus, circ_0013958 positively regulated the PLXNB2 expression by sponging miR-637.

### Depletion of circ_0013958 Hampered OC Tumor Growth *In vivo*

**Figure 7:**
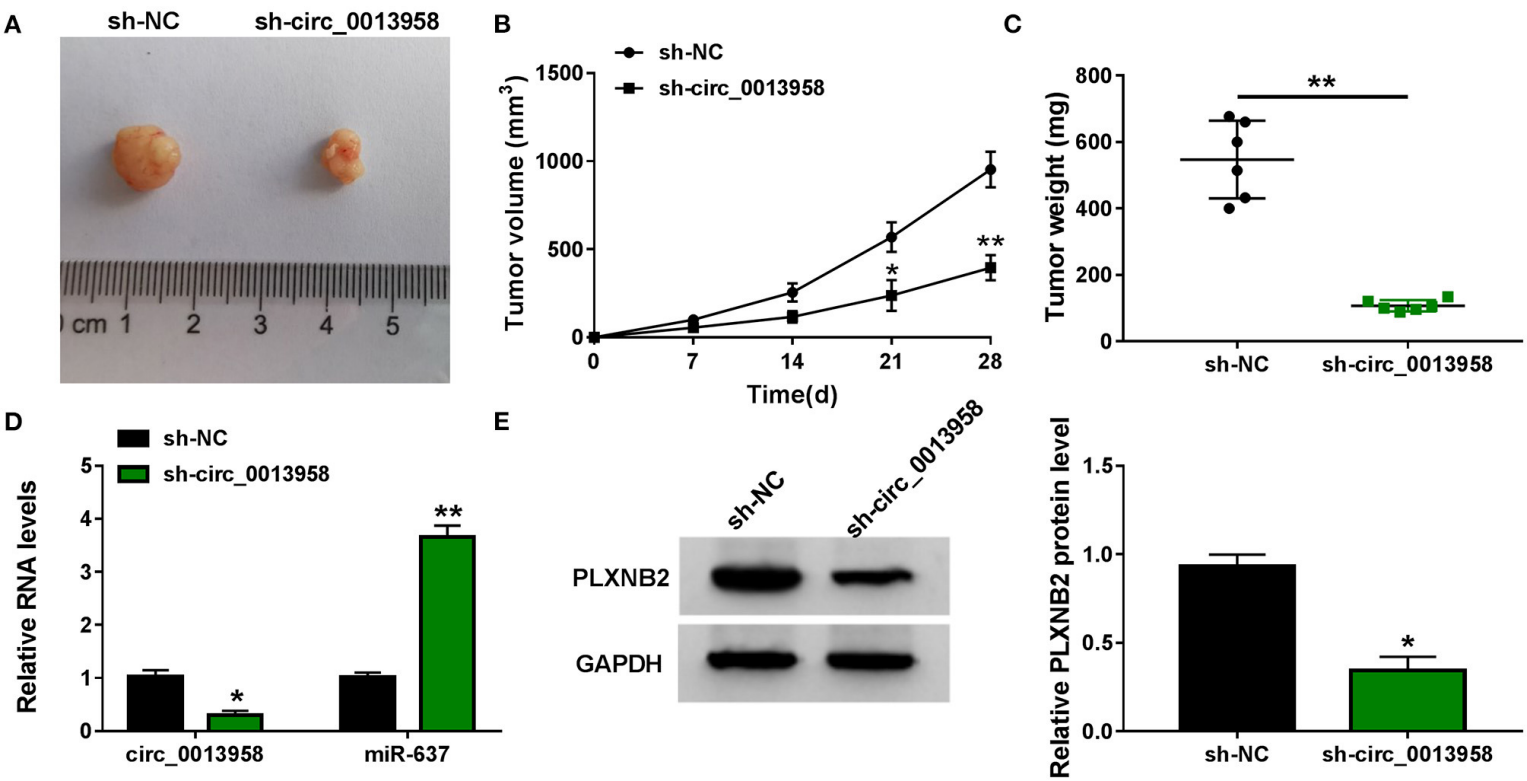
Circ_0013958 silencing hampered OC tumor growth *in vivo*. SKOV3 cells stably expressing sh-NC or sh-circ_0013958 were subcutaneously injected into BALB/c nude mice (*n* = 6). **(A)** Representative images of xenograft tumor tissues. **(B)** Tumor volume was measured every 7 days for 28 days. **(C)** Tumor weight was measured at 28-day post injection. **(D)** QRT-PCR analysis for the expression of circ_0013958 and miR-637 in xenograft tumor tissues. **(E)** Western blot assay for the protein level of PLXNB2 in xenograft tumor tissues. *P < 0.05; **P < 0.01.

A xenograft tumor model was constructed by subcutaneous inoculation with SKOV3 cells stably expressing sh-NC or sh-circ_0013958 into BALB/c nude mice. Compared with the sh-NC group, tumors generated in the sh-circ_0013958 group exhibited smaller volume (Figures 7A,B) and lighter weight (Figure 7C). In addition, the circ_0013958 expression and the PLXNB2 protein level were downregulated, while miR-637 was upregulated in tumors of sh-circ_0013958 in contrast to those of the sh-NC group (Figures 7D,E). Furthermore, the IHC staining assay for PLXNB2 indicated that circ_0013958 silencing significantly depressed the PLXNB2 positive cells in contrast with the sh-NC group (Supplementary Figure 4). Therefore, circ_0013958 knockdown inhibited OC tumor growth *in vivo*.

## Discussion

CircRNAs were substantiated to endow with potential importance in cancer diagnosis and treatment, and could serve as tumor-promoting or suppressing agents (Yin et al., 2019; Yang et al., 2020). Numerous studies have disclosed the dysregulated circRNAs in tumors and their close correlation with aggressive development of OC. For example, circ-001567 was significantly upregulated in OC tissues, and circ-001567 knockdown repressed OC cell proliferation, invasion, and tumorigenicity (Bao et al., 2019). Luo et al. (2018) found that circ-ITCH was downregulated in OC cells, and overexpression of circ-ITCH suppressed OC cell proliferation by antagonizing miR-10a. CircPLEKHM3 was identified to be downregulated in OC tissues, and it acted as a tumor suppressor in OC by sponging miR-9 to inactivate AKT1 and Wnt/β-catenin signaling pathways (Zhang L. et al., 2019). A recent research has disclosed that circ_0013958 was upregulated in OC tissues and cells, and promoted cell proliferation and metastasis in OC (Pei et al., 2020). In accordance with previous research, in this research, we demonstrated that circRNA circ_0013958 was highly expressed in OC tissues and cells. Interestingly, the area under the curve (AUC) was 0.8806, which indicates the good diagnostic value of circ_0013958 in OC. Functionally, knockdown of circ_0013958 inhibited cell proliferation, migration, and invasion but promoted apoptosis in OC *in vivo*. And the xenograft OC tumor model in nude mice indicated that circ_0013958 silencing curbed the OC tumor growth *in vivo*. These results uncovered that circ_0013958 functions as a tumor promoter in OC, which is vital to the tumorigenesis and progression of OC.

It is generally accepted that circRNAs could exert their functions by sponging the target miRNAs and deregulate its suppression effect on downstream targets, thereby participating in tumor initiation and progression regulation (Hansen et al., 2013). Here, miR-637 was predicted to be a target of circ_0013958. As previously reported, miR-637 was downregulated in liver cancer tissues, and it could impede proliferation and invasion of liver cancer cells by targeting AKT1, serving as a cancer-suppressing agent (Du and Wang, 2019). Furthermore, the tumor-suppressor role of miR-637 was also validated in melanoma (Zhang et al., 2018), pancreatic ductal adenocarcinoma (PDAC) (Xu et al., 2018), and colorectal cancer (Wang L. et al., 2019). In current research, circ_0013958 was mainly distributed in the cytoplasm, suggesting that circ_0013958 may act as miRNA sponges. As confirmed by the dual-luciferase reporter assay and the RIP assay, miR-637 harbored complementary binding sites, and circ_0013958 negatively interacted with miR-637 by acting as a sponge for miR-637. Besides, miR-637 was downregulated in OC tissues and cells and played a repression function in OC by regulating cell proliferation, migration, invasion, and apoptosis. Moreover, miR-637 deficiency partly attenuated the suppression effects of circ_0013958 knockdown on OC cell proliferation, migration, invasion, and apoptosis. Taken together, circ_0013958 mediated OC cell proliferation, motility, and apoptosis through sponging miR-637 to decrease the abundance of miR-637 in the cytoplasm.

We also investigated the downstream target of miR-637 using TargetScan software. We found that PLXNB2 was a target of miR-637 and was negatively regulated by miR-637. As the receptors for semaphorins, plexins could modulate the development of the nervous system, cardiovascular system, skeleton, and kidney, as well as affect the functions of the immune system and tumor progression (Perälä et al., 2012). It was reported that PLXNB2 and semaphorin 4C participated in the regulation of the vascular system and endocrine system (Zielonka et al., 2010). Furthermore, the decreased level of PLXNB2 was correlated to unfavorable prognosis of breast cancer patients (Malik et al., 2015). In addition, PLXNB2 could serve as a prognostic marker and drug target for malignant glioma (Le et al., 2015). Xiang and Cheng (2018) alleged that PLXNB2 was a target mRNA of miR-126-3p in OC cells, and PLXNB2 mitigated the miR-126-3p-induced inhibitory impacts on OC cell proliferation and invasion. In our research, the mRNA and protein levels of PLXNB2 in OC tissues and cells were significantly elevated in contrast with adjacent normal tissues. Furthermore, overexpression of PLXNB2 partly counteracted the repression effect of miR-637 mimic on OC cell proliferation, migration, invasion, and apoptosis, indicating the promotion function of miR-637 in OC progression. Additionally, we found that the PLXNB2 expression in OC tissues was positively correlated with the expression of circ_0013958. And the suppression effect of circ_0013958 knockdown on the protein level of PLXNB2 was partly reversed by the miR-637 inhibitor. Moreover, circ_0013958 silencing enhanced the level of miR-637 and decreased the mRNA and protein levels of PLXNB2 in xenograft tumor tissues, thereby curbing the growth of OC tumor *in vivo*. Consequently, circ_0013958 upregulated the expression of PLXNB2 by sponging miR-637 to decrease the abundance of miR-637 in OC.

In conclusion, circ_0013958 was significantly upregulated in OC tissues and cells. Depletion of circ_0013958 repressed cell proliferation, migration, and invasion but promoted apoptosis in OC cells *in vitro*. Circ_0013958 curbed the growth of OC tumors *in vivo*. Mechanistically, circ_0013958 functioned as a sponge for miR-637 to elevate PLXNB2 expression. Our findings uncovered the function of the circ_0013958/miR-637/PLXNB2 axis in OC progression, highlighting a promising target for OC treatment.

## Data Availability Statement

The raw data supporting the conclusions of this article will be made available by the authors, without undue reservation.

## Ethics Statement

The studies involving human participants were reviewed and approved by The Second Nanning People's Hospital. The patients/participants provided their written informed consent to participate in this study. The animal study was reviewed and approved by The Second Nanning People's Hospital.

## Author Contributions

YL designed and performed the experiments, interpreted the results, and revised the manuscript. KM designed and performed the experiments, interpreted the results, and wrote the manuscript. RQ designed the research, interpreted the results, and revised the manuscript. All authors contributed to the article and approved the submitted version.

## Conflict of Interest

The authors declare that the research was conducted in the absence of any commercial or financial relationships that could be construed as a potential conflict of interest.

## Supplementary Material

Supplementary Figure 1 The expression of circ_0013958 in OC cells. Circ_0013958 expression in OC cell line (SKOV3, CAOV3, A2780, and OVCAR-3) and human ovarian surface epithelial cells (HOSE). ^**^*P* < 0.01.

Supplementary Figure 2 The protein level of PLXNB2 in OC tissues. PLXNB2 protein level in OC tissues and adjacent normal tissues. ^**^*P* < 0.01.

Supplementary Figure 3 Correlation between PLXNB2 and circ_0013958 levels in OC tissues. Pearson's correlation analysis for the correlation between the expression levels of circ_0013958 and PLXNB2 in OC tissues (*n* = 30).

Supplementary Figure 4 PLXNB2 level in xenograft tumor tissues with sh-NC or sh-circ_0013958. Immunohistochemistry (IHC) for PLXNB2 in xenograft tumor tissues with sh-NC or sh-circ_0013958. ^**^*P* < 0.01.
