# Supplementary figures and images for: Circular RNA Circ_0013958 Functions as a Tumor Promoter in Ovarian Cancer by Regulating miR-637/PLXNB2 Axis
Source: Front Genet. 2021 Jul 21;12:644451. doi: 10.3389/fgene.2021.644451 (PMC8334736; doi:10.3389/fgene.2021.644451)

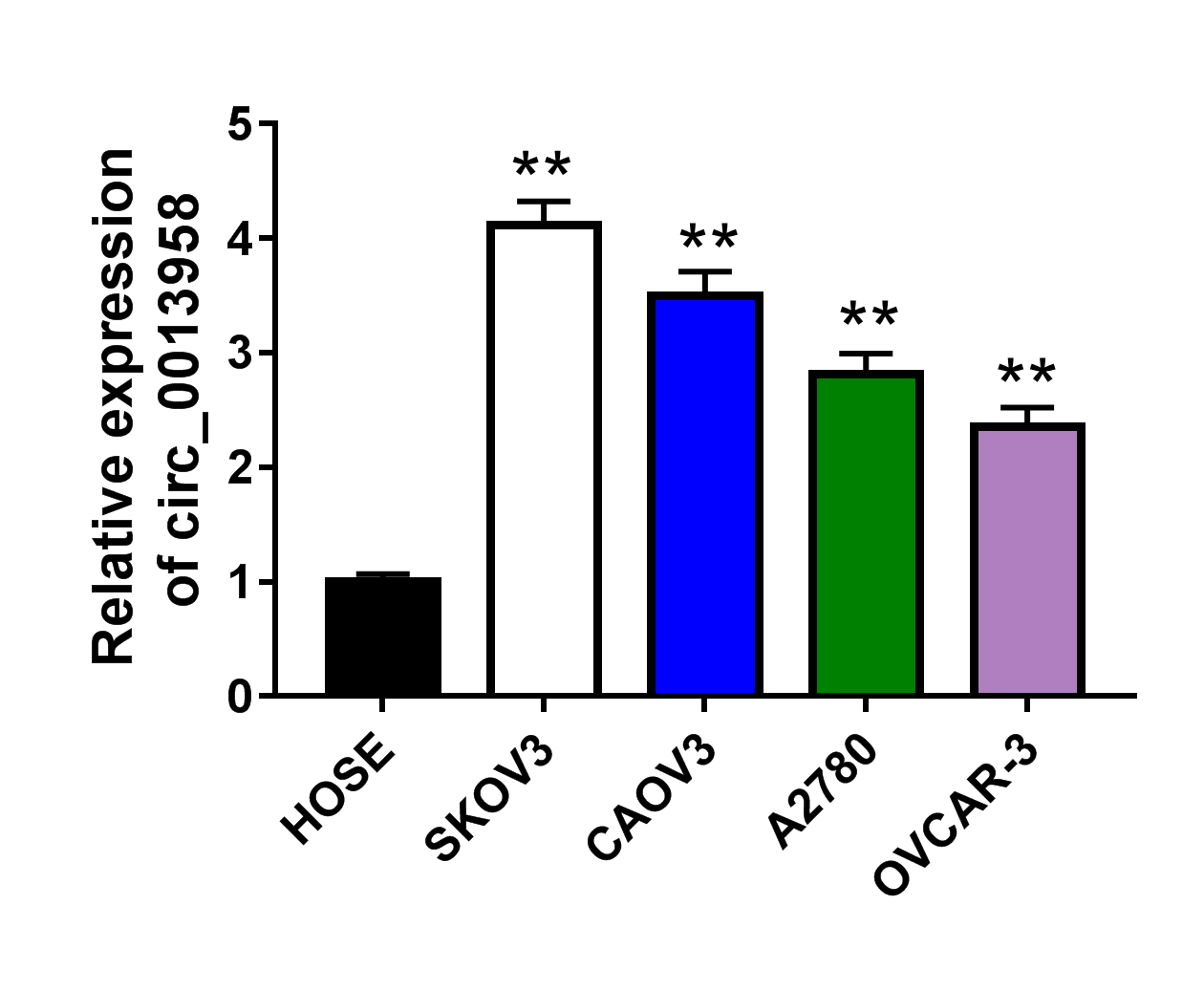

Supplement: Supplementary Figure 1 — The expression of circ_0013958 in OC cells. Circ_0013958 expression in OC cell line (SKOV3, CAOV3, A2780, and OVCAR-3) and human ovarian surface epithelial cells (HOSE). **P < 0.01. [file Image_1.TIF]

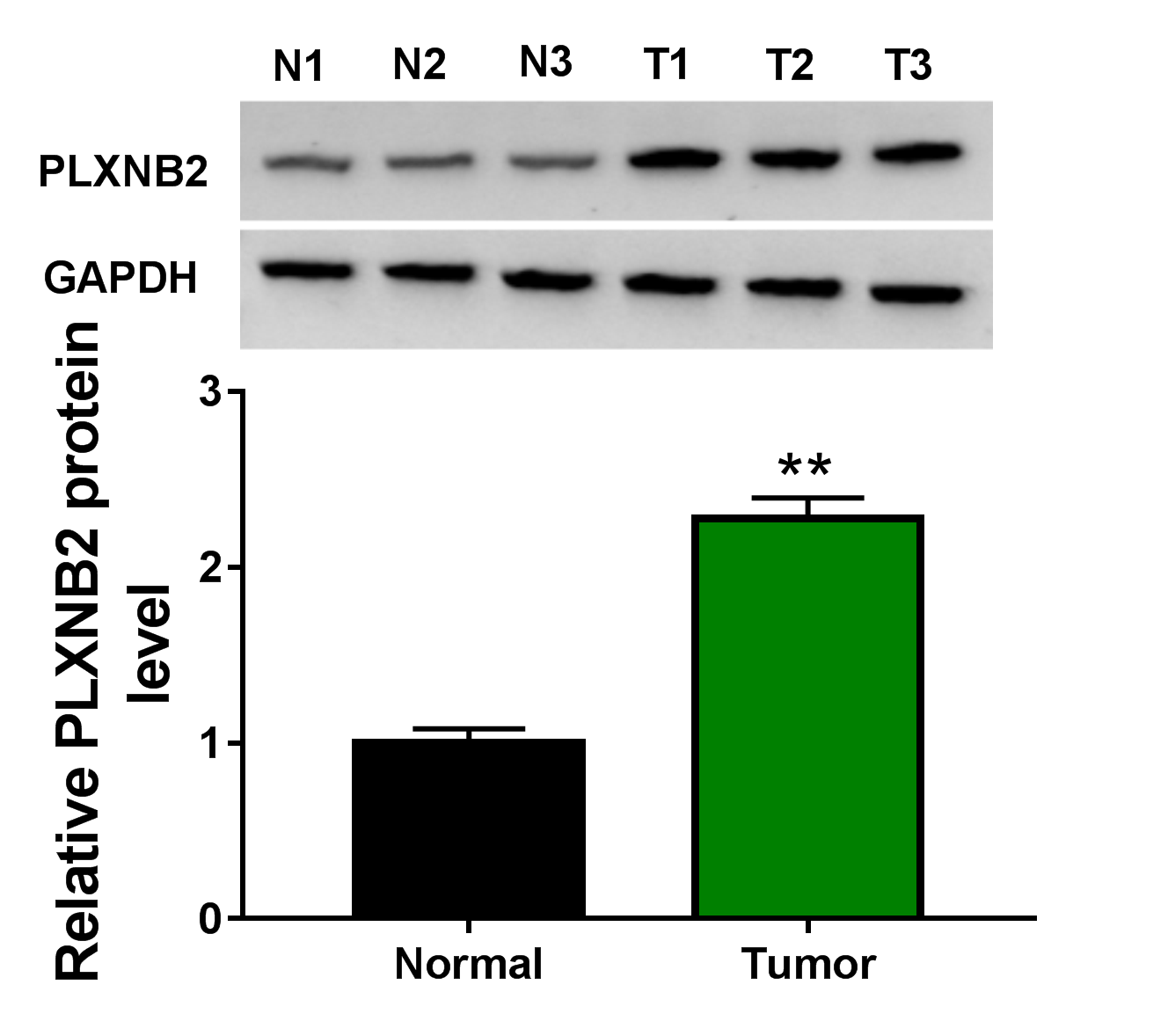

Supplement: Supplementary Figure 2 — The protein level of PLXNB2 in OC tissues. PLXNB2 protein level in OC tissues and adjacent normal tissues. **P < 0.01. [file Image_2.TIF]

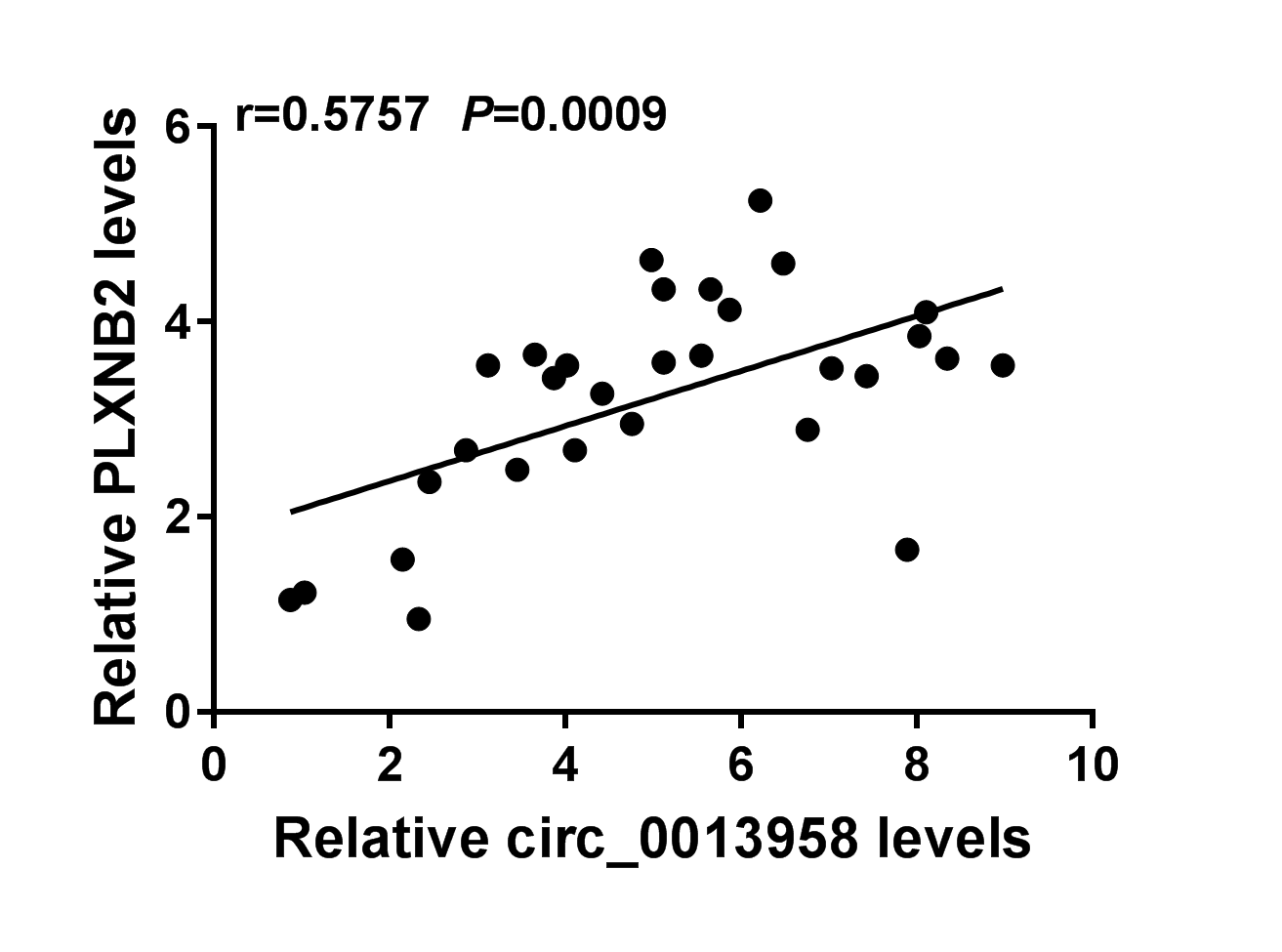

Supplement: Supplementary Figure 3 — Correlation between PLXNB2 and circ_0013958 levels in OC tissues. Pearson's correlation analysis for the correlation between the expression levels of circ_0013958 and PLXNB2 in OC tissues (n = 30). [file Image_3.TIF]

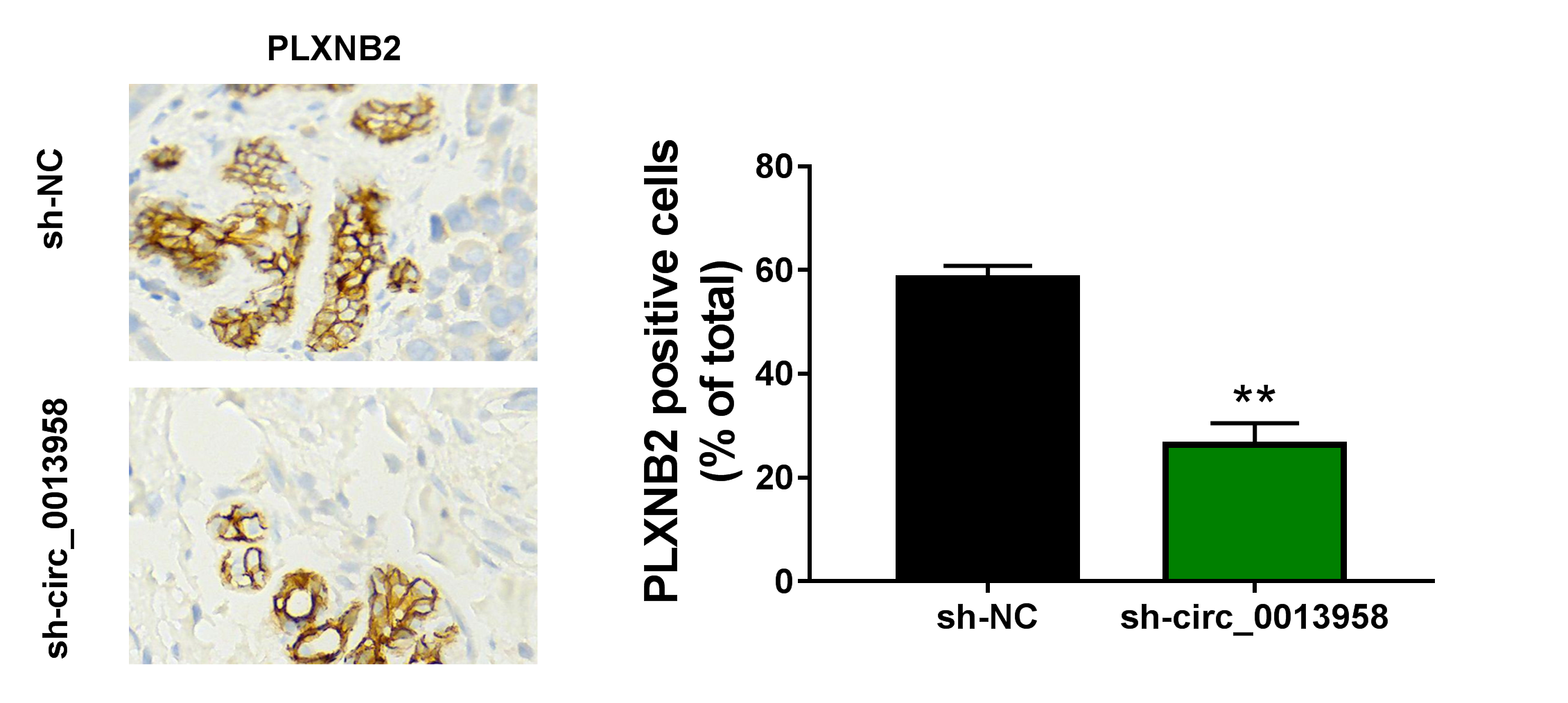

Supplement: Supplementary Figure 4 — PLXNB2 level in xenograft tumor tissues with sh-NC or sh-circ_0013958. Immunohistochemistry (IHC) for PLXNB2 in xenograft tumor tissues with sh-NC or sh-circ_0013958. **P < 0.01. [file Image_4.TIF]
